# Fully automated convolutional neural network-based affine algorithm improves liver registration and lesion co-localization on hepatobiliary phase T1-weighted MR images

**DOI:** 10.1186/s41747-019-0120-7

**Published:** 2019-10-26

**Authors:** Kyle A. Hasenstab, Guilherme Moura Cunha, Atsushi Higaki, Shintaro Ichikawa, Kang Wang, Timo Delgado, Ryan L. Brunsing, Alexandra Schlein, Leornado Kayat Bittencourt, Armin Schwartzman, Katie J. Fowler, Albert Hsiao, Claude B. Sirlin

**Affiliations:** 1 Liver Imaging Group, Department of Radiology, University of California San Diego, La Jolla, CA USA; 2 AiDA Laboratory, Department of Radiology, University of California San Diego, La Jolla, CA USA; 3 Department of Radiology, Stanford University, Palo Alto, CA USA; 4 Abdominal and Pelvic MRI, Radiology, CDPI Clinics, DASA Company, Fluminense Federal University (UFF), Rio de Janeiro, Brazil; 5 Department of Family Medicine and Public Health, University of California San Diego, La Jolla, CA USA; 6 Altman Clinical Translational Research Institute, 9452 Medical Center Drive, Lower Level 501, La Jolla, CA 92037 USA

**Keywords:** Gadolinium ethoxybenzyl DTPA, Image processing (computer assisted), Liver, Magnetic resonance imaging, Neural networks (computer)

## Abstract

**Background:**

Liver alignment between series/exams is challenged by dynamic morphology or variability in patient positioning or motion. Image registration can improve image interpretation and lesion co-localization. We assessed the performance of a convolutional neural network algorithm to register cross-sectional liver imaging series and compared its performance to manual image registration.

**Methods:**

Three hundred fourteen patients, including internal and external datasets, who underwent gadoxetate disodium-enhanced magnetic resonance imaging for clinical care from 2011 to 2018, were retrospectively selected. Automated registration was applied to all 2,663 within-patient series pairs derived from these datasets. Additionally, 100 within-patient series pairs from the internal dataset were independently manually registered by expert readers. Liver overlap, image correlation, and intra-observation distances for manual *versus* automated registrations were compared using paired *t* tests. Influence of patient demographics, imaging characteristics, and liver uptake function was evaluated using univariate and multivariate mixed models.

**Results:**

Compared to the manual, automated registration produced significantly lower intra-observation distance (*p* < 0.001) and higher liver overlap and image correlation (*p* < 0.001). Intra-exam automated registration achieved 0.88 mean liver overlap and 0.44 mean image correlation for the internal dataset and 0.91 and 0.41, respectively, for the external dataset. For inter-exam registration, mean overlap was 0.81 and image correlation 0.41. Older age, female sex, greater inter-series time interval, differing uptake, and greater voxel size differences independently reduced automated registration performance (*p* ≤ 0.020).

**Conclusion:**

A fully automated algorithm accurately registered the liver within and between examinations, yielding better liver and focal observation co-localization compared to manual registration.

**Electronic supplementary material:**

The online version of this article (10.1186/s41747-019-0120-7) contains supplementary material, which is available to authorized users.

## Key points

- Image registration across series can improve lesion co-localization and reader confidence
- Combining convolutional neural network-based segmentation with affine transformations created a fully automated three-dimensional registration method for magnetic resonance images of the liver
- This algorithm improved liver overlap and focal liver observation co-localization over standard manual registration

## Background

Characterization of focal liver observations (*i.e.,* areas distinctive from background liver representing either lesions or pseudolesions) [1] requires synthesis of imaging features across multiple contrast-enhanced phases and/or series and often demands incorporation of data across exams acquired at multiple time points. Proper spatial alignment during and between exams is challenged by the dynamic morphology of the liver and variability in patient positioning, body habitus, and physiological motion [2–4]. For instance, differences in respiratory phase may shift the liver position by as much as 30 mm between acquired images [5–8]. Such shifts can significantly reduce radiologists’ ability to co-localize observations across series, especially when there are multiple observations and/or exams [9–11].

Most radiologists in clinical practice rely on manual, and rigid registration of images (*i.e.,* scrolling through a stack of images to find the most similar slice position for comparison) acquired at different time points, which can be time-consuming and achieves partial alignment only in the slice direction. Alignment across the entire liver volume is not possible in routine clinical practice, and separate alignments may be required for each observation. The need for repeated alignment is tedious, slows workflow, introduces opportunities for alignment errors, and potentially contributes to interpretive mistakes. By making images acquired at different time points, positions, or modalities geometrically similar, image registration can improve observation co-localization and reader confidence [12, 13].

Affine and deformable liver-focused medical image registration algorithms have been proposed to address these challenges but typically require operator supervision and are slowed by intensive processing and computing requirements [14–16]. Also, most algorithms have been evaluated only in small patient cohorts without generalization and have not been adopted in clinical radiology practice [10, 11, 17–19].

Convolutional neural networks (CNN) have been used in deformable registration tasks [20–24] and can potentially overcome some of these barriers. However, since the nonlinear transformations of deformable registration can cause anatomical distortions, affine registration algorithms (which restrict image transformations to scaling, rotation, translation, and shearing) are generally preferred for diagnostic imaging [25, 26]. Studies have shown that liver registration is improved by first segmenting the liver to create a liver mask and then using the mask rather than the whole image as input into the registration algorithm [14, 17]. CNNs can provide the required segmentation and potentially improve the accuracy of the liver registration. We combined these technical advances to create a fast, fully automated 3D affine registration algorithm for liver imaging that incorporates CNN-based liver segmentation.

The purpose of this study was to assess the performance of the proposed fully automated 3D affine algorithm to register intra- and inter-exam liver imaging series and to compare its performance to standard manual image registration. Secondary purposes were to confirm that incorporating CNN-based liver segmentation improves registration performance, to compare registration performance using different types of CNN-segmented liver masks, and to show scalability and generalizability of the results. As an exploratory aim, we performed cross-modality (CT to MRI) and multiphase (arterial phase to hepatobiliary phase MRI) registration as proof of concept.

## Methods

### Design

**Fig. 1:**
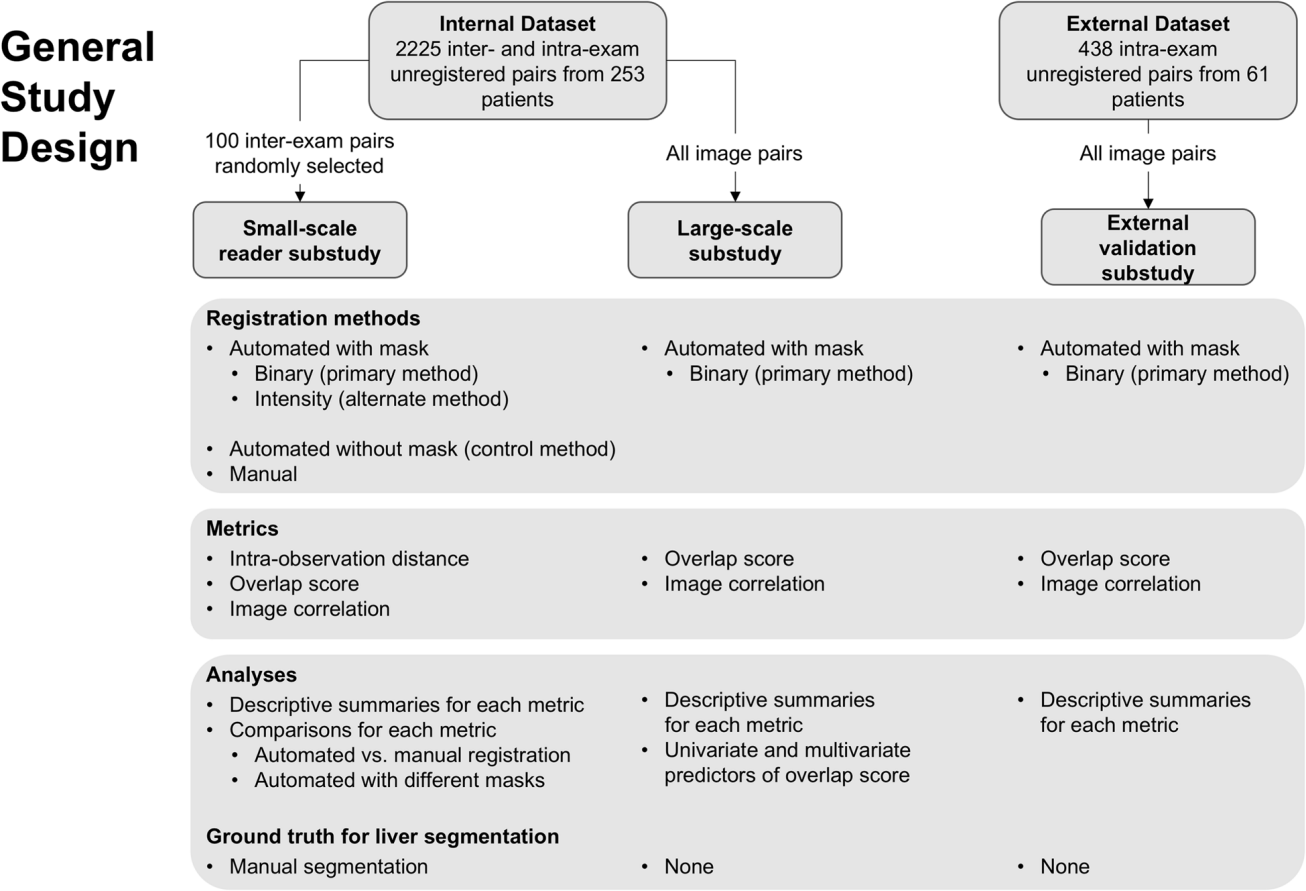
Study design

In this retrospective dual-center study, we included gadoxetate disodium-enhanced three-dimensional (3D) T1-weighted hepatobiliary phase (HBP) magnetic resonance imaging (MRI) studies in adult patients for clinical care. A subset of images from an internal dataset was used to conduct a small-scale reader substudy, which compared the performance of the algorithm to manual registration performed by expert radiologists. The complete set of images from the internal dataset was used to show algorithm scalability and identify factors that affect registration performance (large-scale substudy). An external dataset was used to show algorithm generalizability (external validation substudy). The overall study design is shown in Fig. 1. The study was Health Insurance Portability and Accountability Act compliant and approved by the institutional review board. Informed consent was waived.

### Image data

The internal image dataset comprised images from patients with chronic liver disease undergoing two or more gadoxetate-enhanced liver MRI examinations at 1.5 or 3 T (General Electric Medical Systems, WI, USA) for hepatocellular carcinoma surveillance at our institution from 2011 to 2018. These images were 3D fat-saturated T1-weighted gradient-echo series, acquired 15 min or later after contrast injection (HBP images). The contrast agent was intravenously administered at the dose of 0.025 mmol/kg. Baseline and follow-up studies of 253 unique patients provided a total of 564 unique HBP series because some patients had multiple HBP series acquired at different delays after contrast injection in a given examination. All inter-exam series pairs (different examinations) and intra-exam pairs (different HBP series acquired within the same examination) produced a total of 2,225 possible within-patient series pairs. Table 1 summarizes patient and imaging characteristics.

**Table 1:** Demographic and imaging characteristics of the 253 patients in the internal dataset as well as their effects on liver overlap score

	Demographic summary		Univariate analysis			Multivariate analysis		
Stratum	Number of image pairs	Number of patients	Coefficient	95% CI	*p*	Coefficient	95% CI	*p*
Overall	2,225	253	0.86	[0.85, 0.87]	< 0.001			
Age* (years)	58.38 ± 12.37 (38.60–77)							
< 45 (intercept)	260	33	0.89	[0.85, 0.92]	< 0.001	/	/	/
45–64	1,211	142	− 0.02	[− 0.06, 0.02]	0.34	− 0.01	[− 0.04, 0.02]	0.56
> 65	754	78	− 0.05	[− 0.09, − 0.01]	0.02	− 0.04	[− 0.07, 0.00]	0.06
Sex								
M (intercept)	1,062	108	0.87	[0.86, 0.89]	< 0.001	/	/	/
F	1,163	145	− 0.03	[− 0.05, 0.00]	0.02	− 0.03	[− 0.05, − 0.01]	0.01
BMI* (kg/m^2^)	20.06 ± 5.99 (20.65–39.53)							
< 24.9 (intercept)	571	62	0.83	[0.81, 0.86]	< 0.001			
25–30	792	91	+ 0.03	[− 0.00, 0.06]	0.05			
> 30	842	94	+ 0.04	[0.01, 0.07]	0.01			
Etiology								
Hepatitis B (intercept)	466	37	0.85	[0.82, 0.89]	< 0.001			
Hepatitis C	1,042	108	+ 0.01	[− 0.02, 0.05]	0.50			
Alcohol	164	33	− 0.01	[− 0.06, 0.04]	0.63			
Nonalcoholic fatty liver disease	276	35	+ 0.02	[− 0.02, 0.07]	0.39			
Autoimmune hepatitis	98	11	+ 0.03	[− 0.03, 0.10]	0.34			
Other	179	29	+ 0.00	[− 0.05, 0.05]	0.93			
Time between series* (days)	324.72 ± 407.73 (0–1,255)							
Same day (intercept)	674	196	0.9	[0.89, 0.92]	< 0.001	/	*/*	*/*
0–1 year	823	135	− 0.05	[− 0.06, − 0.04]	< 0.001	− 0.03	[− 0.04, − 0.02]	< 0.001
1–2 years	469	93	− 0.08	[− 0.09, − 0.07]	< 0.001	− 0.05	[− 0.07, − 0.03]	< 0.001
> 2 years	259	29	− 0.10	[− 0.12, − 0.08]	< 0.001	− 0.08	[− 0.10, − 0.06]	< 0.001
Artifacts								
No artifacts (intercept)	1,278	185	0.86	[0.85, 0.88]	< 0.001			
One image	597	105	− 0.00	[− 0.02, 0.01]	0.68			
Both images	350	65	− 0.01	[− 0.02, 0.01]	0.55			
HBP contrast uptake adequacy								
Both adequate (intercept)	1,435	193	0.87	[0.86, 0.88]	< 0.001	/	/	/
One adequate, one suboptimal	350	54	− 0.02	[− 0.03, − 0.01]	0.01	− 0.02	[− 0.03, 0.00]	0.01
Both suboptimal	340	62	+ 0.02	[− 0.01, 0.04]	0.15	+ 0.00	[− 0.02, 0.02]	0.96
Missing	100	16	/	/	/	/	/	/
Voxel volume difference* (mm^3^)	0.76 ± 1.52 (0–3.02)							
0 (intercept)	746	186	0.90	[0.89, 0.92]	< 0.001	/	/	/
0–1	1,064	141	− 0.06	[− 0.07, − 0.05]	< 0.001	− 0.02	[− 0.03, − 0.01]	< 0.001
> 1	402	86	− 0.09	[− 0.10, − 0.07]	< 0.001	− 0.05	[− 0.06, − 0.03]	< 0.001
Slice thickness* (mm)	4.53 ± 0.73 (3.00–6.00)							
Pixel spacing* (mm)	0.82 ± 0.15 (0.70–0.94)							

*BMI* body mass index, *HBP* hepatobiliary phase

* Mean +/- standard deviation; 5th and 95th percentiles in parentheses

As part of another study [27], series in the internal dataset were reviewed by two abdominal fellowship-trained radiologists (A.H. and G.M.C.), with 5 and 10 years of experience in liver imaging, who independently determined the presence of imaging artifacts and additionally classified each series as A (adequate HBP contrast uptake) or B (suboptimal HBP contrast uptake). Series were classified according to the Liver Imaging Reporting and Data System [1] as adequate if the liver was unequivocally more hyperintense than hepatic blood vessels and suboptimal if this criterion was not met. Discordant classifications were adjudicated in consensus.

The external image dataset comprised T1-weighted 3D fat-saturated gradient-echo HBP acquisitions from patients who underwent gadoxetate disodium-enhanced 1.5-T liver MRI examinations (Siemens Healthcare, Erlangen, Germany) for various clinical indications at an outpatient imaging center outside the USA (Diagnósticos da América SA [DASA]) from September to November 2018. A predosed syringe with 10 ml (0.25 mmol/ml) of gadoxetate disodium-based contrast was infused by peripheral IV at a rate of 1 ml/s. This dataset included intra-exam series pairs only, since full anonymization of patient information prior to data transfer precluded tracking exams acquired at different dates to the same individual. Sixty-one unique patients provided 192 unique HBP series with 438 possible within-patient series pairs. Patient characteristics for this cohort were not available.

For the exploratory aim, we selected by convenience from the internal dataset one patient (a 47-year-old male) with contemporaneous contrast-enhanced computed tomography (CT) and one patient (a 78-year-old female) with arterial-phase 3D T1-weighted images acquired in the same exam as the HBP images.

### Fully automated affine registration algorithm

The registration algorithm comprised a previously developed liver segmentation CNN [28] and an affine transformation network executed on a workstation with a Titan V graphics processing unit (NVIDIA, CA, USA) and implemented on the Keras Application Program Interface (API) [29], a widely used open-source toolkit for deep neural network implementation.

For inter-exam registration, the HBP series from the baseline exam served as the “static” series while the HBP series from the follow-up exam served as “moving” series. For intra-exam registration, the earlier HBP series after contrast injection served as the “static” series whereas the later HBP series served as “moving” series. For the exploratory aim, the CT series or the arterial-phase MR series served as the moving series.

**Fig. 2:**
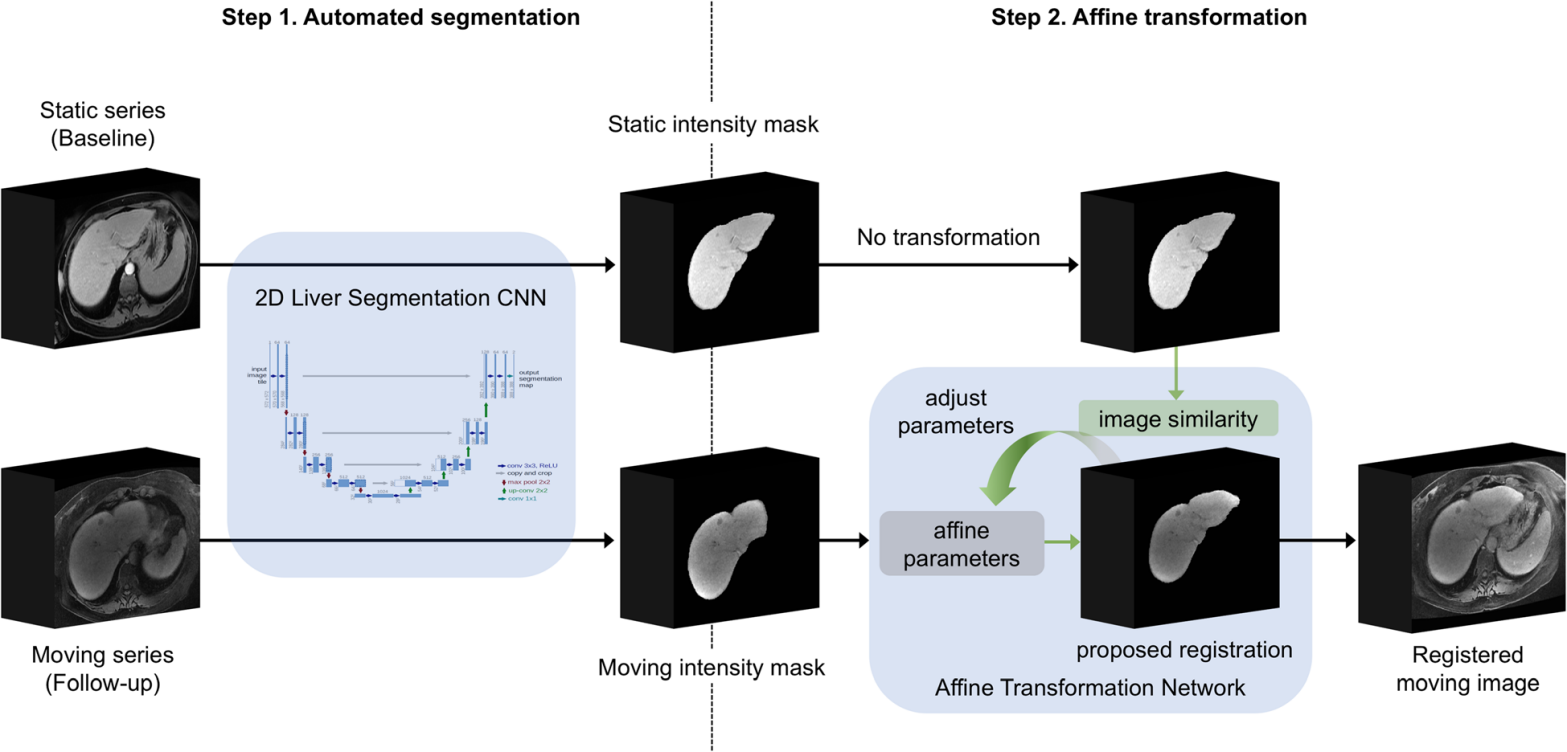
Scheme of the two-step fully automated affine registration algorithm using intensity masks. First, an independently developed two-dimensional liver segmentation algorithm was used to extract liver masks populated with intensities. Intensity masks were registered using an affine transformation network to geometrically align the moving series (follow-up) to the static series (baseline). Optimal affine transformation parameters were determined by maximizing the similarity between baseline and registered follow-up. Affine transformation parameters were then used to map the entire moving series to the static image series space

Automated registration of each pair of static and moving series was accomplished in two steps (Fig. 2). First, the CNN segmented the liver on each series to create liver masks. Second, the liver masks were registered using the affine transformation network. The affine transformation parameters were then applied to the whole images, not just the liver masks, to map the moving series to the static series space. Both the liver segmentation CNN and affine transformation network are described further below.

#### Convolutional neural network for liver segmentation

The liver segmentation model is a two-dimensional CNN with U-Net model architecture trained to segment T1-weighted HBP images and previously validated using liver volumetry and proton density fat fraction estimations [28]. Axial slices from 3D HBP series were individually sent through the liver segmentation network to produce a set of liver masks (one per slice), which were then concatenated and post-processed to form a 3D binary liver mask. For additional details on the development and implementation of the liver segmentation CNN, please refer to Additional file 1: S1. The resulting masks were binary: pixels within the mask had values of 1 and pixels outside the mask had values of 0. Automated registration using the “binary masks” was considered the primary investigational method, since it used native spatial resolution and, lacking any pixel intensity information, has the potential for generalization to multimodality or multiphase registration. A variant of the binary masks called “intensity mask” was then created by multiplying the 3D binary mask by the original raw liver image, thereby populating the masks with the signal intensities from the corresponding image; this was done to investigate how the additional pixel intensity information affected registration performance. Each of the two masks (binary and intensity) was tested as input for the affine transformation network. As a control, the affine transformation was also tested using whole acquired images without any liver segmentation; this was done to determine the effect of focusing the registration on the liver masks on registration accuracy.

#### Affine transformation network

The affine transformation was implemented as a neural network with a single 12-neuron dense layer representing 3D affine transformation parameters for translation, rotation, scaling, and shearing. The network estimated affine transformation parameters that optimized alignment between the moving liver mask (*i.e.,* binary or intensity mask) and the static liver mask. Using these transformation parameters, the original, unmasked moving series was transformed to the static series space. A similar process was used for registering whole images (*i.e.,* without segmentation), except all transformations and mappings were done using moving and static whole images rather than moving and static masks. For additional details on the implementation of the affine transformation network, please refer to Additional file 1: S1.

### Small-scale reader substudy

**Fig. 3:**
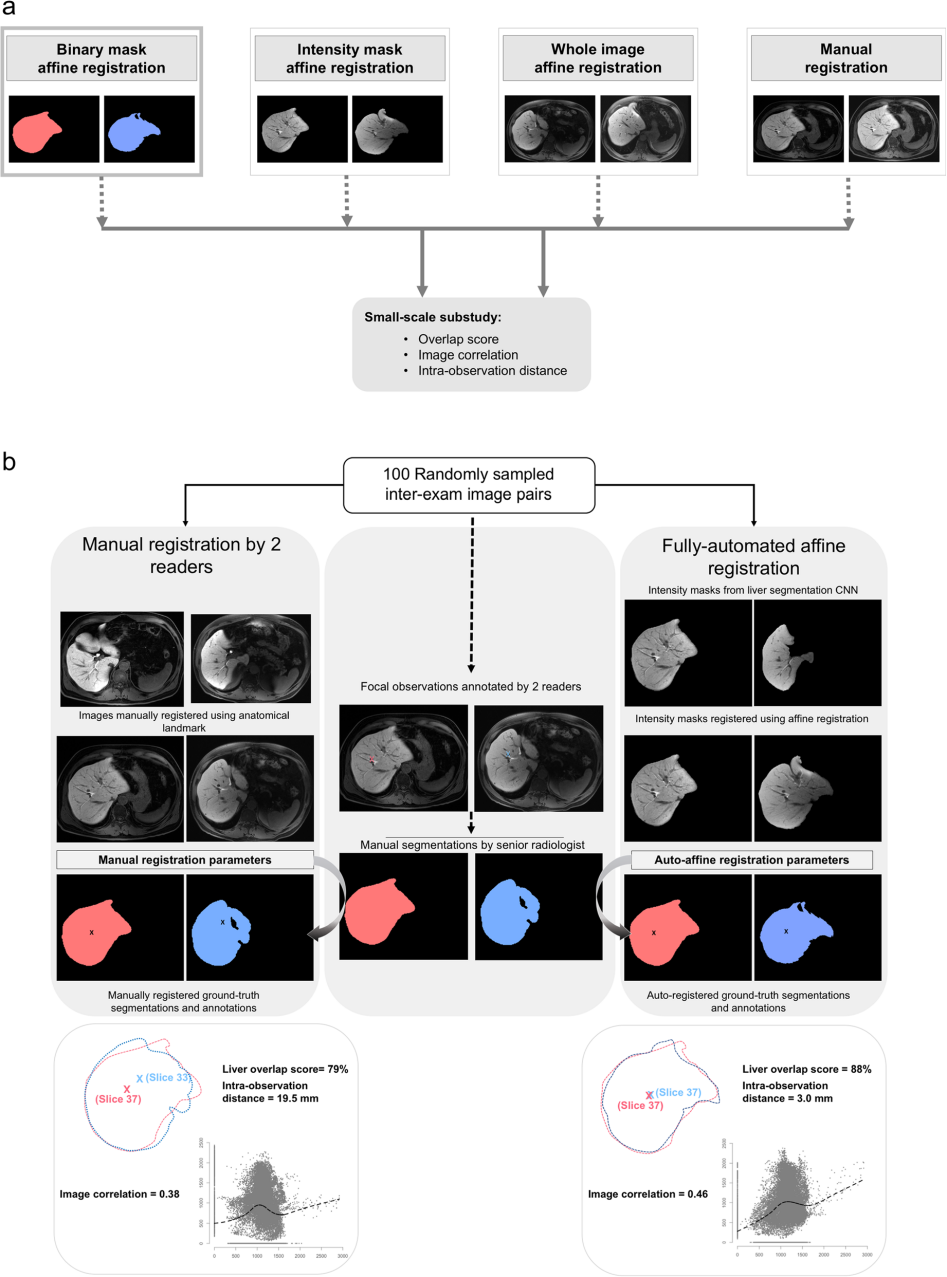
Small-scale substudy (**a**): Registration methods and performance metrics. Binary mask (first, left to right) was considered the primary investigational method for registration and it was compared to alternate masking method, *i.e.,* whole image registration and manual registration. Small-scale reader substudy (**b**): flow and performance comparisons between radiologist-performed manual registration and automated algorithm (intensity masks displayed). Three metrics: liver overlap score (Jaccard index), image correlation (mutual information), and intra-observation distance

To evaluate how the automated algorithm with binary mask compares to radiologist-performed manual registration and to automated registration with different masks or no masks, we randomly selected 100 inter-exam series pairs from the internal imaging dataset and compared performance using three metrics (Fig. 3).

#### Manual registration of static and moving series

Two expert readers (fellowship-trained body radiologists [A.H. and S.I.], each with 5 years of experience in liver imaging), independently, manually registered 100 series pairs (whole images) in the *z*-direction using a commercially available DICOM viewer (Osirix, Geneva, Switzerland) to reflect common clinical practice. Readers registered the images by aligning slices depicting the right portal vein bifurcation in both series as an internal landmark. Slice numbers corresponding to the aligned slices were recorded for each series for subsequent analyses described below.

#### Manual annotation of focal liver observations on static and moving series

In the same reading session, the two readers independently annotated any detected focal liver observation on each of the 100 series pairs (static and moving series, separately). Usually, these were distinctive hypointense round nodules. Each annotation included a marker indicating the spatial coordinates of the observation center. After observations were annotated in all image pairs, readers measured the single smallest and largest ones to obtain observations size range.

#### Fully automated affine registration

The automated registration algorithm (using mask variations and whole images) was applied to the 100 inter-exam series pairs. Affine transformation parameters from the 100 automated registrations were saved for use in subsequent analyses.

#### Manual liver segmentation to create ground-truth liver masks

A senior radiologist (G.M.C.) with 10 years of experience in liver imaging manually segmented the livers on the same 100 series pairs (static and moving series, separately) to create ground-truth liver masks. As described below, these ground-truth liver masks were used to compare the accuracy of automated *versus* manual registration.

#### Comparison of manual and automated registration

Each radiologist’s observation annotations were overlain on the ground-truth liver masks, preserving their exact coordinates, to create “annotated masks.” To create auto-registered series pairs, the saved affine transformation parameters were applied to the “annotated moving masks.” To create manually registered series pairs, the annotated masks were aligned in the *z*-direction using the slice positions selected by each reader as described above. The moving masks were then scaled, rotated, and translated (rigid transformations) to correct for differences in field-of-view or patient positioning from the static masks. This was done to maximize the accuracy of manual registration in the performance comparisons below. Three metrics were used to assess and compare the accuracy of the manual and automated registrations: (1) Liver overlap score (Jaccard index) [30] between the static and registered moving liver masks over the entire liver volume (a score of 1 indicates perfect liver overlap); (2) Image correlation (mutual information) [31] between the ground-truth static intensity mask and the registered ground-truth moving intensity mask (a larger image correlation indicates better registration performance; of note, since image correlation relies on the pixel intensity distribution of the series for calculation, we used image correlation to make only paired comparisons, *i.e.,* we would have been unable to determine if differences in image correlation were attributed to algorithm performance or pixel distributional differences between unpaired groups); and (3) 3D intra-observation distance (marker of focal liver observation co-localization) in millimeter between matched observation centers on overlain static and registered moving series (a score of 0 mm indicates perfect observation co-localization; in addition to quantitative metrics, a separate analysis qualitatively comparing manual registration and binary mask registration using reader confidence scores for image similarity assessment was performed. Details on this analysis are described in Additional file 1: S2.

### Large-scale and external validation substudies

Since the intensity mask was not meaningfully superior to the binary mask in the small-scale reader study (see results) and since binary masks were considered the primary investigational method, the subsequent studies focused on binary masks. The automated registration algorithm (using binary masks) was applied to all 2,225 within-patient series pairs in the internal dataset to evaluate scalability and to all 438 intra-exam series pairs in the external dataset to evaluate generalizability. Two metrics were used to evaluate registration accuracy: (1) Liver overlap score and (2) image correlation. Intra-observation distance was not assessed as it was not feasible to manually annotate individual observations on the large number (*n* = 2,663) of series in these studies. Similarly, since ground-truth manual segmentation was not feasible for all series pairs, liver overlap score and image correlation were calculated using the masks produced by the CNN liver segmentation algorithm. Computation time was recorded.

### Feasibility of cross-modality and multiphase registration

The registration algorithm (using binary masks) was applied to a single cross-modality series pair (CT to MRI) and a single intra-exam series pair (HBP to arterial phase MRI) as proof-of-concept examples of cross-modality and multiphase registration, respectively. After registration, liver overlap score (Jaccard index) [30] between the static and registered moving liver masks over the entire liver volume was calculated.

### Statistical analysis

All statistical analyses were performed by a biostatistician using R v3.4.0 software [32]. Descriptive summaries were prepared. In the small-scale reader study, the effect of HBP adequacy on liver segmentation accuracy (liver overlap score) was evaluated using linear mixed effects models. Registration metrics achieved by automated registration with the binary mask (the primary investigational method) was compared using paired Bonferroni-corrected *t* tests to those achieved by every other method (manual registration by each reader, automated registration with intensity mask, and automated registration without any mask [whole image]). Additionally, readers were compared to each other. In the large-scale study, intra-exam and inter-exam differences were compared using unpaired *t* tests. Influence of patient demographics, inter-series time intervals, imaging artifacts, reader consensus-determined HBP adequacy, and voxel volume differences on one registration metric (liver overlap score) achieved by the primary automated registration algorithm (binary masks) was evaluated in univariate and multivariate analyses, using linear mixed effects models to account for within-patient dependencies. Significant characteristics in the multivariate analysis were determined by backward elimination. Ninety-five percent confidence intervals (CIs) were analytically calculated as appropriate. The *p* values lower than 0.05 were defined as significant for *t* tests and linear mixed effects models.

## Results

### Small-scale reader substudy

**Fig. 4:**
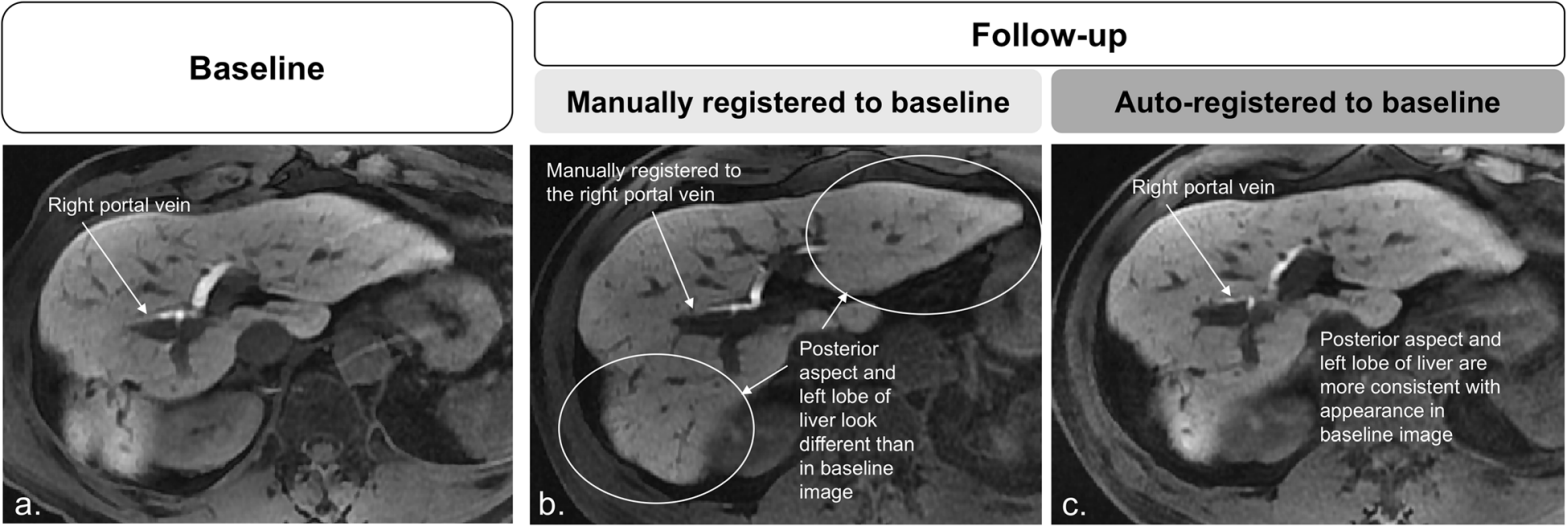
Baseline static (**a**) and follow-up moving images (**b**, **c**). In **b**, the follow-up image registered to baseline manually using the bifurcation of the right portal vein as anatomical reference: differences in liver morphology are pronounced in the posterior aspect of the liver (circle) and in the left lobe (circle). In **c**, the follow-up image registered to baseline using the automated affine algorithm shows better correspondence to baseline image

**Fig. 5:**
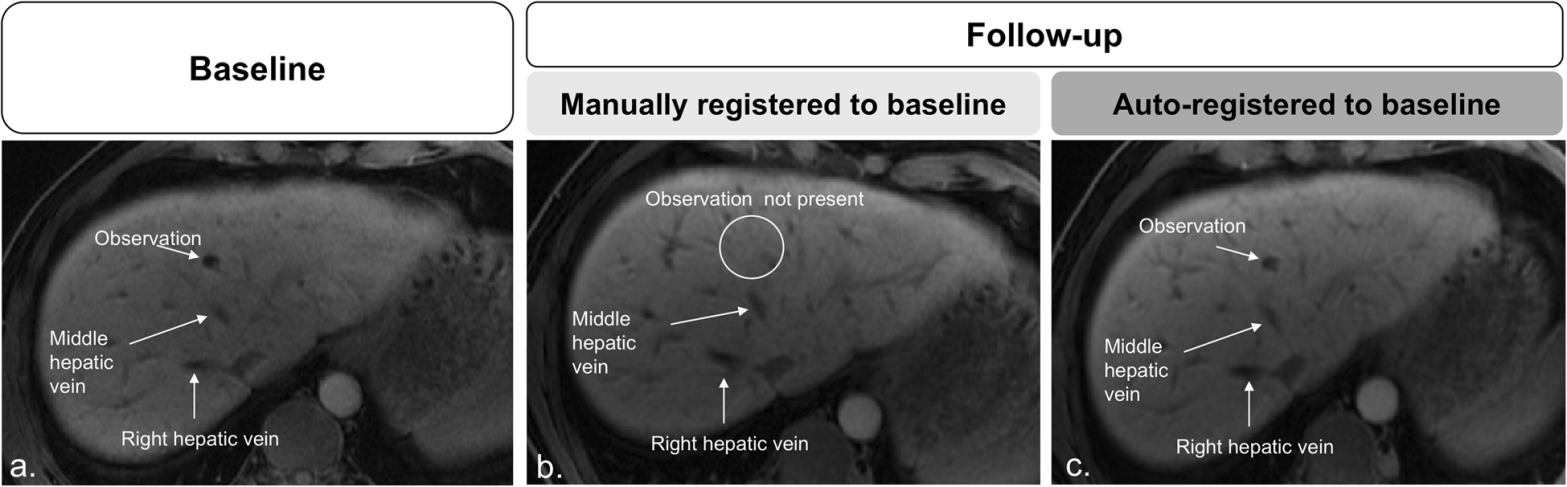
Manual registration of baseline static (**a**) and follow-up moving (**b**, **c**) images show aligned hepatic veins. A 1.0-cm focal liver observation is seen in segment IVa in baseline (**a**) but not seen on follow-up image (circle) (**b**). In **c**, follow-up registered image using the automated affine algorithm parameters applied to the whole image shows correspondence to the baseline image, including the presence of the same focal observation in segment IVa.

**Fig 6:**
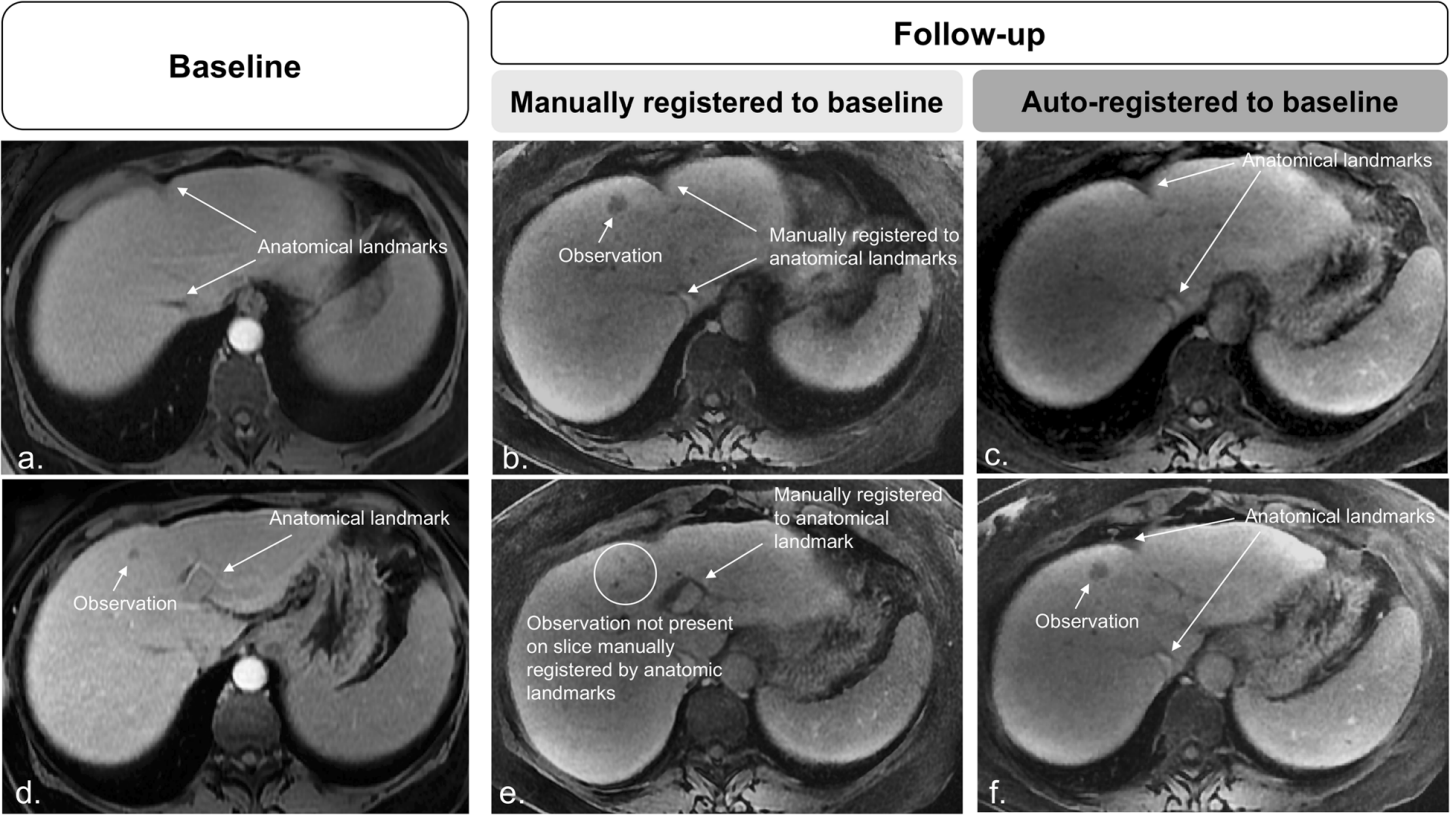
Baseline (**a**, **d**) and follow-up (**b**, **c**, **e**, and **f**) images. Manual registrations show the alignment of slices through anatomical references. However, focal observations are noted in different slice positions. The mismatch between focal observations and slice position negatively affects assessment as the observation in **d** may be interpreted as a new lesion. **c**, **f** On the automated affine registered follow-up images, focal liver observation correspondence is confirmed, and the diagnosis of previous existing growing lesion is made with higher confidence

Performance metrics (mean ± standard deviation) for the automated and manual registrations in the small-scale study are summarized in Table 2; paired mean differences and their Bonferroni-corrected *p* values are listed in Additional file 1: Table S1. Figures 4, 5 and 6 provide examples of higher qualitative spatial concordance on auto-registered series over manually registered series.

**Table 2:** Mean ± standard deviation of performance metrics across 100 within-patient series pair for manual and automated registrations in the small-scale substudy

Registration method		Liver overlap score	Image correlation	Observation distance (annotated by reader 1)	Observation distance (annotated by reader 2)
Manual registration	Reader 1	0.74 ± 0.10	0.36 ± 0.14	14.73 ± 8.20	/
	Reader 2	0.73 ± 0.12	0.35 ± 0.14	/	16.09 ± 8.49
Automated registration (with masking)	Binary mask	0.86 ± 0.06	0.42 ± 0.16	8.40 ± 4.62	8.42 ± 5.78
	Intensity mask	0.86 ± 0.06	0.43 ± 0.16	8.26 ± 4.64	8.49 ± 5.67
Automated registration (without masking)	Whole image	0.73 ± 0.15	0.39 ± 0.17	22.95 ± 24.42	18.11 ± 16.06

Statistical comparisons between registration methods were performed using paired *t* tests (Additional file 1: Table S1)

#### Liver segmentation accuracy

Mean liver overlap score between predicted and ground-truth liver masks was 0.96 ± 0.04. Liver overlap scores were not significantly different between series with adequate and suboptimal HBP contrast uptake (*p* = 0.88).

#### Liver overlap score

On manually registered series, mean liver overlap score was 0.73 or 0.74, depending on the reader; these differences were not significant. Compared to manual registration, automated registration with binary mask improved the mean liver overlap score from 0.73–0.74 by 0.11–0.12 to 0.86, depending on the reader. In pairwise comparisons, binary mask and intensity mask showed similar overlap scores (mean liver overlap 0.86) and were not statistically different. Automated registration without the initial liver segmentation (*i.e.,* using whole images) was inferior (mean liver overlap 0.73) to binary mask (adjusted *p* < 0.001) and did not improve mean liver overlap score compared to manual registration.

#### Image correlation

On manually registered series, mean image correlation was 0.35 or 0.36, depending on the reader; these differences were not significant. Compared to manual registration, automated registration with binary mask improved the mean image correlation from 0.35–0.36 by 0.06–0.07 to 0.42, depending on the reader. In pairwise comparisons, binary mask yielded statistically lower image correlation than intensity mask (0.42 *versus* 0.43, adjusted *p* < 0.001). Automated registration with whole images provided significantly lower image correlation than automated registration using binary masks (0.39 *versus* 0.42, adjusted *p* < 0.001).

#### Observation distance

One reader detected and annotated 139 observations (range 2–68 mm), and the other reader 147 observations (range 2–70 mm). On manually registered series, mean observation distances were 14.7 mm and 16.1 mm, depending on the reader; these differences were not significant (*p* = 0.47). Compared to manual registration, automated registration with binary mask decreased the mean intra-observation distance by 6.3–7.7 to 8.4 mm, depending on the reader. In pairwise comparisons, binary mask was not statistically or meaningfully superior to any other mask. Automated registration without a mask (*i.e.,* using whole images) provided mean liver observation distances of 18 and 23 mm; these values were significantly inferior to automated registration with binary mask (*p* < 0.001).

The automated registration algorithm improved qualitative readers’ confidence scores for image similarity over standard manual registration as described in Additional file 1: Figure S1**.**

### Large-scale and external validation substudies

Liver overlap and image correlation means ± standard deviations achieved by automated registration with a binary mask in the large-scale and external validation studies are shown in Table 3. For the large-scale study, mean liver overlap score was 0.81 for inter-exam registration and 0.88 for intra-exam registration while mean image correlation was 0.41 for inter-exam registration and 0.44 for intra-exam registration; differences between intra-exam and inter-exam registrations were significant (*p* < 0.001). For the external validation study, mean liver overlap score was 0.91 and mean image correlation was 0.41. Mean computation time for inter-exam binary mask registration (54.6 s) was significantly longer than intra-exam binary mask registration (50.3 s) (*p* < 0.001).

**Table 3:** Performance analysis (mean ± standard deviation) of all within-patient pair combinations (2,225) of hepatobiliary phase images in the internal and external datasets (438 image pairs)

Dataset	Liver overlap score	Image correlation
Internal dataset (intra-exam)	0.88 ± 0.14	0.44 ± 0.15
Internal dataset (inter-exam)	0.81 ± 0.14	0.41 ± 0.14
External dataset (intra-exam)	0.91 ± 0.06	0.41 ± 0.13

Statistical comparisons between internal datasets were performed using unpaired *t* tests

Table 1 summarizes the influence of patient factors, inter-series time intervals, image artifacts, and inter-series voxel volume differences on liver overlap achieved by automated registration with a binary mask in the internal dataset. In multivariate analyses, older age, female sex, greater inter-series time interval, and greater inter-series voxel volume differences independently reduced the liver overlap score. Additionally, liver overlap score was reduced when registering two series with different HBP contrast uptake adequacy scores.

### Feasibility of cross-modality and multiphase registration

**Fig. 7:**
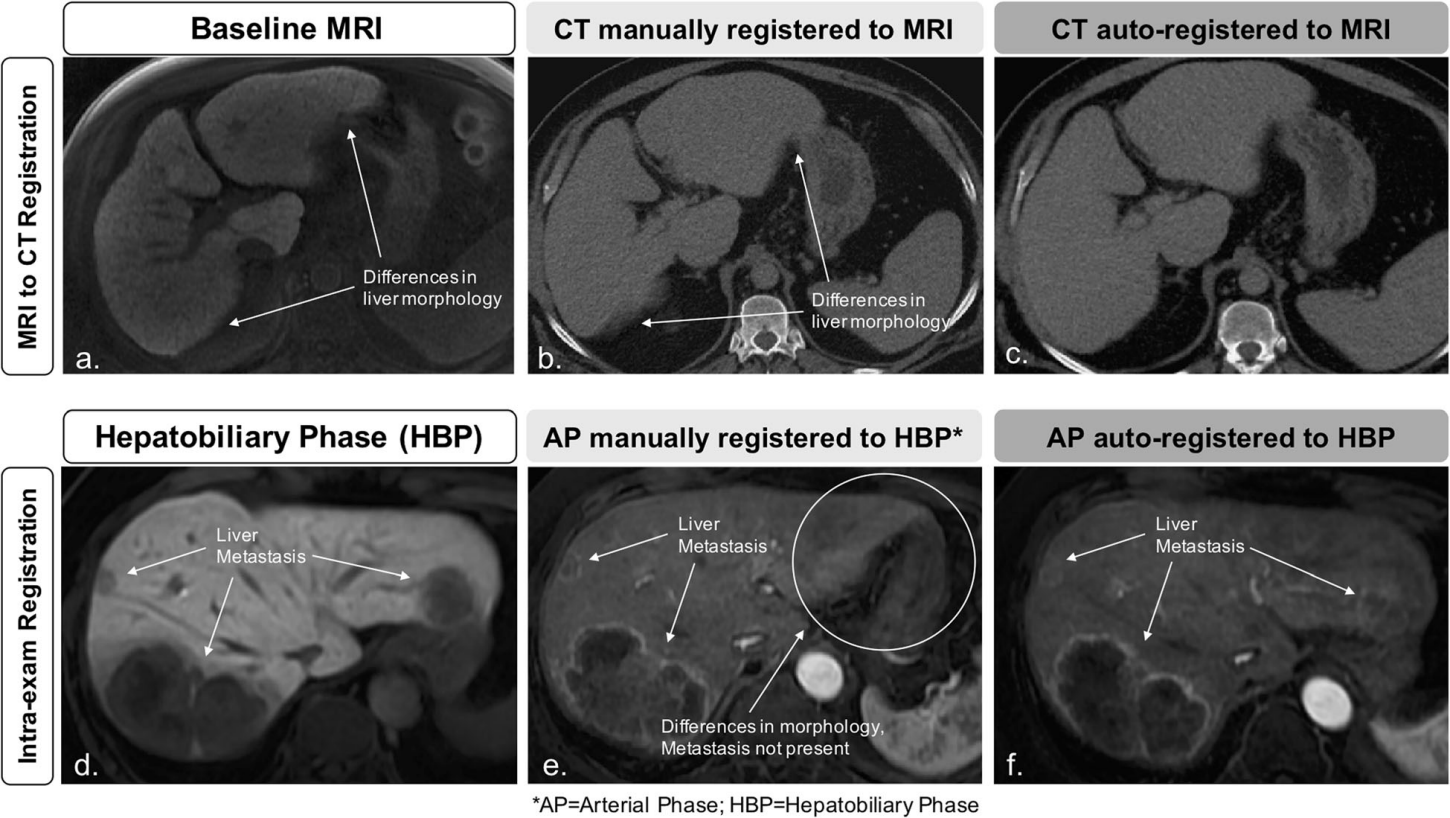
Feasibility of cross-modality and multiphase registration. The registration algorithm (using binary masks) was applied to a cross-modality series pair (computed tomography to magnetic resonance imaging; **a**–**c**) and one intra-exam multiphase series pair (HBP to arterial phase; **d**–**f**) as proof-of-concept examples of cross-modality and multiphase registration, respectively

Feasibility of cross-modality and multiphase registration using binary masks is shown in Fig. 7. Cross-modality (CT to MRI) liver overlap score was 0.93 and multiphase (arterial phase to hepatobiliary phase MRI) liver overlap score was 0.89 using the proposed automated method.

## Discussion

In this study, we applied a novel fully automated affine-based registration algorithm to MRI T1-weighted HBP series acquired at different delays from contrast injection within the same exam and at different exam dates and compared its performance against standard manual registration. The automated algorithm produced better registration of the liver between series over manual registration and, most importantly, better co-localization of focal observations.

In clinical practice, image registration is commonly performed manually, which confines the registration to magnification and to alignment in the *z*-direction. This means that the radiologist may be able to scroll up or down, zoom in or out, to find the slice that most closely aligns to the comparison image, but cannot alter the image in any other plane. Rotational alignments can be performed using multiplanar reformation software add-ons, often time-consuming and difficult to perform. Our proposed 3D automated registration saves time and mitigates cross-sectional misalignment attributed to changes in position or dynamic liver morphology between series, as the CNN-based liver segmentations/masks focus the affine transformations to the organ of interest, which, as shown by others and verified in this study, improves accuracy [14].

Compared to masks that retained intensity information, binary masks achieved overall similar performance, except for small and likely unmeaningful differences in image correlation, suggesting that the liver masking (not the liver intensities) drives registration accuracy. Since the affine transformation does not meaningfully benefit from intensity information, the proposed algorithm using binary masks does not rely on a particular imaging sequence, phase, or modality and can potentially be extended to cross-modality and multiphase registration tasks. Finally, we showed the proposed algorithm is scalable and generalizable across a large number of exams and different populations and scanners, achieving high liver overlap scores and image correlations across over 2,500 series pairs.

The performance of the proposed automated registration algorithm was not affected by patient body mass index, liver disease etiology, and imaging artifacts. Inter-exam registration performance was significantly affected by the time between series; compared to intra-exam series, average liver overlap was reduced by 10% when images were acquired more than 2 years apart. This was expected since the liver may change in volume and shape over prolonged time periods, especially in patients with chronic liver disease [33, 34]. Differences in voxel volume between series also reduced registration performance, probably because such differences force the 3D registration to interpolate the moving series across a different anisotropic space than the static image. Differences in HBP contrast uptake adequacy within a series pair reduced registration performance, but the reduction was small and likely not clinically relevant. We believe this minor decrease in registration performance could be related to small differences in liver mask predictions between adequate and suboptimal series, where small isointense vessels in suboptimal series may be included in the liver masks. Registration performance was not affected when both series in a pair were either adequate or suboptimal. Older age and female sex each mildly decreased registration accuracy. We speculate that different breath-hold capabilities or breathing patterns across age and sex [35] could affect variability in liver position and shape between acquisitions. Further research is needed to confirm and elucidate the mechanism for these findings.

The incorporation of segmentation techniques to constrain registration focus on the organ of interest and improve performance over whole image registration has been proposed [4, 9, 14, 18, 19]. However, these often relied on laborious manual or semiautomated segmentation and/or were validated on small cohorts [9, 11, 14, 17]. Being completely automated, and hence, less time-intensive than semiautomated or manual methods, our registration algorithm can be applied to a large set of image series pairs while achieving greater accuracy than whole image registration (*i.e.,* without segmentation). Additionally, most prior works proposed deformable techniques to address the nonlinear morphology of the liver [9, 10, 17, 18]. Although achieving good performance overall, deformable registration can be unreliable and inadvisable for diagnostic purposes since distortions in organ or lesion appearance may lead to erroneous measurements or characterization [13, 25, 26]. Hence, some investigators favor non-deformable image registration for lesion evaluation and follow-up [25, 26]. Carrillo et al. [11] and Fujioka et al. [19] proposed rigid registration of liver images to evaluate locoregional therapy response with promising results, reporting mean registration errors ranging from 3.05 to 13.00 mm. Although similar to the intra-observation distances reported in our study, results were reported on small cohorts (< 20 patients) and relied on extensive image preprocessing, manual registration, and/or manual liver segmentations, possibly limiting application to busy clinical settings [11, 19]. Additionally, Carrillo et al. [11] reported the exclusion of slices close to the diaphragm in the reference volumes as well as the exclusion of 2 out of 17 individuals due to low-image quality (low signal-to-noise ratio [SNR] or motion artifacts). Overall, our algorithm was not affected by the presence of imaging artifacts, likely due to the robustness of our liver segmentation algorithm.

Foruzan and Motlagh [14] have used a multistep approach to register liver images for interventional therapy purposes. In their study, a semiautomated liver segmentation was followed by a rigid and a non-rigid registration. Their liver segmentation achieved slight lower overlap (0.93) than our CNN-based liver segmentation. For their rigid and non-rigid registration algorithms, mean liver overlap and intra-observation distance were 0.75 and 11.7 mm and 0.78 and 10.11 mm, respectively. These values are slightly worse than those achieved by our algorithm. Although the robustness of our CNN-based liver segmentation may in part be the reason why we found slightly better results, their study performed multimodality registration on low-field strength MR data with very low SNR images, making their registration task more challenging.

Conversely, Fernandez-de-Manuel et al. [36] proposed a liver-focused deformable registration algorithm using high SNR gadoxetate disodium-enhanced 3D T1-weighted HBP images for liver lesion evaluation. In their study, they reported a mean intra-observation distance of 7.07 mm after registration, which is similar to our results. However, since they used a deformable registration technique, a direct comparison to our results may warrant some caution. Unfortunately, we could not find any publicly available datasets or CNN-segmentation-based algorithms for liver registration to test our approach and/or perform a head-to-head comparison. Our proposed algorithm also achieved shorter computation times than previously proposed liver registration algorithms, which ranged from under 2 min [14] to 2 to 30 min [8, 9]. However, faster computation times may be attributed to technological advances in computing (using graphics processing units) and differences in the registration task (affine *versus* deformable). Hence, it is likely the computation time reported in our study will be easily overcome as technology advances in the next few years.

A limitation of our study was its retrospective design, which precluded the assessment of how our algorithm would impact radiologist performance in a clinical setting. Additionally, our cohort was comprised mostly of patients with chronic liver disease and under surveillance for HCC. Hence, the number of patients with advanced neoplastic disease, locoregional treatment, or liver resections was small. Therefore, we could not assess how major alterations in liver size and shape related to these procedures would impact the registration. Finally, since ground-truth manual segmentations were not feasible for all 2,663 series pairs used in the large-scale and external validation studies, liver overlap score and image correlation were calculated using the masks produced by the CNN liver segmentation algorithm.

In conclusion, our proposed two-step fully automated affine registration algorithm accurately registers the liver within and between examinations and yields significantly better overall liver and focal observation co-localization compared to the manual alignment commonly performed in clinical practice.

## Additional files

Additional file 1: S1. Fully-automated affine registration algorithm. S2. Reader confidence study for qualitative image similarity assessment. **Table S1.** Paired comparisons for the small-scale reader substudy. **Figure S1.** Reader confidence scores for qualitative image similarity assessment. (DOCX 10388 kb)

## Data Availability

The datasets used and/or analyzed during the current study are available from the corresponding author on reasonable request

## Abbreviations

3D: Three-dimensional
CNN: Convolutional neural network
CT: Computed tomography
HBP: Hepatobiliary phase
MRI: Magnetic resonance imaging
