# Supplementary material for: Fully automated convolutional neural network-based affine algorithm improves liver registration and lesion co-localization on hepatobiliary phase T1-weighted MR images
Source: Eur Radiol Exp. 2019 Oct 26;3:43. doi: 10.1186/s41747-019-0120-7 (PMC6815316; doi:10.1186/s41747-019-0120-7)
Supplement: Supplementary file 1 — S1. Fully-automated affine registration algorithm. S2. Reader confidence study for qualitative image similarity assessment. Table S1. Paired comparisons for the small-scale reader substudy. Figure S1. Reader confidence scores for qualitative image similarity assessment. (DOCX 10388 kb) [file 41747_2019_120_MOESM1_ESM.docx]

**Supplementary Materials for “Fully automated convolutional neural network-based affine algorithm improves liver registration and focal observation co-localization on hepatobiliary phase T1-weighted MRI”**

**S1 Fully-automated affine registration algorithm**

The registration algorithm comprises a previously developed liver segmentation CNN [1] and an affine transformation network implemented in Keras [2] and executed on a workstation with a NVIDIA (CA, USA) Titan V graphics processing unit. Automated registration of each pair of static and moving series was accomplished in two steps. First, the CNN segmented the liver on each series to create liver masks. Second, the liver masks were registered using the affine transformation network. The affine transformation parameters were then applied to the whole images, not just the liver masks, to map the moving series to the static series space.

*Convolutional neural network for liver segmentation*

The liver segmentation model is a two-dimensional CNN with U-Net model architecture originally trained to segment the liver on multi-echo spoiled-gradient-echo (SGPR), T1w HBP MR images, and CT images [1]. For this study, the model was fine-tuned with an additional 100 T1w HBP images to further increase segmentation accuracy for this type of image (these images were not included in either dataset used in the analyses). The axial slices from the 3D series were individually sent through the liver segmentation network to produce a set of liver masks (one per slice), which were then concatenated to form a 3D liver mask. 3D liver masks were subsequently refined using binary erosion, dilation, and connected component image processing techniques [3] to produce final binary liver masks.

*Affine transformation network*

The affine transformation was implemented as a neural network with a single 12-neuron dense layer representing 3D affine transformation parameters for translation, rotation, scaling, and shearing. Briefly, the affine transformation network transformed the moving liver mask (i.e., binary or intensity mask) to static space using 12 affine parameters initialized to the identity transformation (no scaling, translation, rotation, or shearing). The transformed moving mask was then compared to the static mask using an image similarity metric (dice coefficient loss for binary masks, normalized cross-correlation loss for intensity masks). Affine parameters were iteratively adjusted with a gradient descent algorithm with Adam stochastic optimizer [4] to improve image similarity between the transformed moving and static masks until convergence. Prior to network optimization, images were down-sampled from native 512x512 resolution to 256x256 resolution to reduce computation time. Upon convergence of affine parameters using the down-sampled images, images were registered at native 512x512 resolution for a reduced number of finer iterations until final convergence. Using the final transformation parameters, the original, unmasked moving series was transformed to the static series space. Since the affine transformation network optimization was performed on each image pair individually without any supervision, there was no need for a leave-out dataset for testing.

**S2 Reader confidence study for qualitative image similarity assessment**

*Design*

Two weeks following the primary reader substudy, to avoid recall bias, the two expert readers reviewed the same 100 image pairs as reading session one, duplicated. These 200 registered series pairs comprised 100 manually registered pairs and 100 affine registered pairs, in random order. Readers were blinded to the registration method and were not informed that any of the series pairs had been previously registered. The readers then manually aligned the baseline and follow-up series on a commercially available DICOM viewer using the right portal vein bifurcation as internal landmark. After aligning the corresponding slices, they assigned a 3-point qualitative score on image and liver anatomical features similarity, as follows: 1 – not confident that features are well colocalized, 2 – slightly confident that features are well colocalized and 3 – very confident that features are well colocalized.

*Statistical Analysis*

Reader confidence scores were compared using Cohen’s kappa for reader agreement. Differences in reader confidence scores between manual and automated registration were analyzed using descriptive statistics

*Results*

Qualitative image similarity scores by both readers are summarized in **Figure S1**. Inter-reader agreement for qualitative image similarity was 0.32, p<0.001. Overall, 73/100 or 84/100 auto-preregistered series pairs were scored as having high spatial concordance, depending on the reader, compared to 40/100 and 46/100 manually registered series. Scores were higher for auto-preregistered series than manually registered series in 32/100 or 49/100 series pairs depending on the reader. Fewer than 5 auto-preregistered series image pairs received lower scores than manual registration by either reader.

*Discussion*

The fully-automated registration algorithm improved reader confidence on image similarity and features colocalization between baseline and follow-up series when compared to standard manual registration.

**References**

1. Wang K, Mamidipalli A, Retson T, Bahrami N, Hasenstab K, Blansit K, Bass E, Delgado T, Cunha G, Middleton MS, Loomba R. Automated CT and MRI Liver Segmentation and Biometry Using a Generalized Convolutional Neural Network. Radiology: Artificial Intelligence. 2019 Mar 27;1(2):180022.
2. Chollet, F et al. Keras. Github, <https://github.com/fchollet/keras>. [Online; accessed 2018-11-07].
3. Jones E, Oliphant E, Peterson P, et al. **SciPy: Open Source Scientific Tools for Python,** 2001, http://www.scipy.org/ [Online; accessed 2019-02-19].
4. Kingma D and Ba J. Adam: A Method for Stochastic Optimization. International conference on learning representations. 2014.

**Table S1.** Paired comparisons for the small-scale reader substudy.

| **Metric** | **Contrast** | **Difference** | **95% CI** | **p-value** | **adjusted  p-value** |
| --- | --- | --- | --- | --- | --- |
| Liver overlap score | Reader 1 - Reader 2 | 0.01 | [-0.01, 0.03] | 0.32 | 1.00 |
|  | Binary mask - Reader 1 | 0.12 | [0.11, 0.14] | <0.001 | <0.001 |
|  | Binary mask - Reader 2 | 0.13 | [0.11, 0.16] | <0.001 | <0.001 |
|  | Binary mask - Intensity mask | 0.00 | [0.00, 0.00] | 0.17 | 1.00 |
|  | Binary mask - Fast mask | 0.01 | [0.01, 0.01] | <0.001 | <0.001 |
|  | Binary mask - Whole image | 0.13 | [0.11, 0.16] | <0.001 | <0.001 |
|  |  |  |  |  |  |
|  |  |  |  |  |  |
| Image correlation | Reader 1 - Reader 2 | 0.00 | [0.00, 0.01] | 0.40 | 1.00 |
|  | Binary mask - Reader 1 | 0.07 | [0.05, 0.08] | <0.001 | <0.001 |
|  | Binary mask - Reader 2 | 0.07 | [0.05, 0.08] | <0.001 | <0.001 |
|  | Binary mask - Intensity mask | -0.01 | [-0.01, 0.00] | <0.001 | <0.001 |
|  | Binary mask - Fast mask | 0.01 | [0.01, 0.01] | <0.001 | <0.001 |
|  | Binary mask - Whole image | 0.03 | [0.02, 0.05] | <0.001 | <0.001 |
|  |  |  |  |  |  |
|  |  |  |  |  |  |
| Observation distance (annotated by reader 1) | Binary mask - Reader 1 | -6.33 | [-8.67, -3.99] | <0.001 | <0.001 |
|  | Binary mask - Intensity mask | 0.14 | [-0.80, 1.08] | 0.77 | 1.00 |
|  | Binary mask - Fast mask | -0.92 | [-1.63, -0.2] | 0.01 | 0.27 |
|  | Binary mask - Whole image | -14.55 | [-22.28, -6.81] | <0.001 | 0.01 |
|  |  |  |  |  |  |
|  |  |  |  |  |  |
| Observation distance (annotated by reader 2) | Binary mask - Reader 2 | -7.67 | [-10.26, -5.08] | <0.001 | <0.001 |
|  | Binary mask - Intensity mask | -0.07 | [-0.99, 0.85] | 0.88 | 1.00 |
|  | Binary mask - Fast mask | -0.39 | [-0.95, 0.17] | 0.17 | 1.00 |
|  | Binary mask - Whole image | -9.69 | [-14.92, -4.46] | <0.001 | 0.01 |
|  |  |  |  |  |  |
|  |  |  |  |  |  |
| Observation distance  (reader difference) | Reader 1 - Reader 2 | -1.36 | [-5.05, 2.33] | 0.47 | 1.00 |
|  |  |  |  |  |  |
| *Shaded rows indicate no significant difference at 5% level* | | | | | |

**
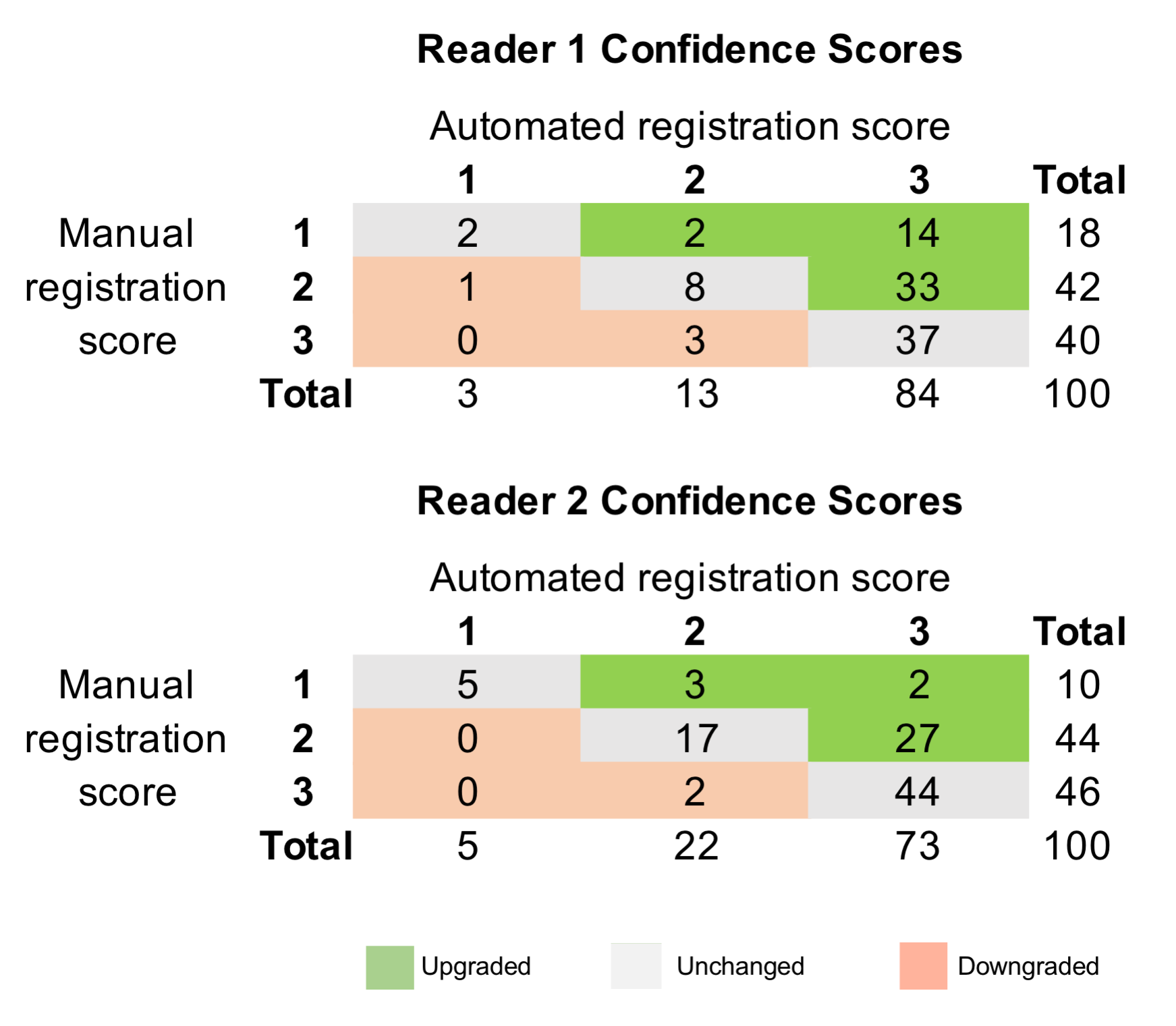
**

**Figure S1:** Reader confidence scores for qualitative image similarity assessment.
